# Characterization and phylogenetic analysis of the chloroplast genome of *Duhaldea cappa* (Buch.-Ham. ex D.Don) Pruski & Anderb. (Asteraceae)

**DOI:** 10.1080/23802359.2024.2306203

**Published:** 2024-01-25

**Authors:** Junjia Luo, Xiaofeng Liu, Tingyu Li, Hui Chen, Tianmeng Qu, Yueguang Wang, Shuhua Yu, Zhixi Fu

**Affiliations:** a Ministry of Education, Key Laboratory of Land Resources Evaluation and Monitoring in Southwest (Sichuan Normal University), Chengdu, China; b College of Life Sciences, Sichuan Normal University, Chengdu, China; c Sustainable Development Research Center of Resources and Environment of Western Sichuan, Sichuan Normal University, Chengdu, China

**Keywords:** Medicinal plant, plastome, phylogenetic relationship, Asteraceae, *Duhaldea cappa*

## Abstract

*Duhaldea cappa,* a valuable medicinal plant of genus *Duhaldea* in the tribe Inuleae, is predominantly found in China, Bhutan, India, Malaysia, Nepal, Pakistan, Thailand, and Vietnam. However, the genomic studies of *Duhaldea cappa* are limited. In this study, we successfully sequenced and assembled the complete chloroplast genome of *Duhaldea cappa*. The chloroplast genome is 150,819 bp in length with a 37.73% GC content. The chloroplast genome has a quadripartite structure, consisting of a large single-copy region of 82,731 bp, a small single-copy region of 18,168 bp, and a pair of inverted repeat sequences of 24,960 bp. The genome contains 133 genes. Among these genes, there are 88 protein-coding genes, 37 tRNA genes, and 8 rRNA genes. The phylogeny reconstructed from data of the complete chloroplast genome indicated that *Duhaldea cappa* is closely related to *Pluchea indica* in the tribe Inuleae. Analyzing and reporting the chloroplast genome of *Duhaldea cappa* will establish a solid theoretical and data foundation for the efficient development, conservation, and utilization of this plant species.

## Introduction

*Duhaldea cappa* (Buch.-Ham. ex D.Don) Pruski and Anderberg (2003) is a valuable medicinal plant, belonging to the genus of *Duhaldea* in the tribe Inuleae, family Asteraceae (Anderberg 1989, Anderberg 2005, Englund et al. 2009, Nylinder and Anderberg 2015). *Duhaldea cappa* is primarily found in Fujian, Guangdong, Guangxi, Guizhou, Hainan, Sichuan, Yunnan, Zhejiang provinces of China, Bhutan, India, Malaysia, Nepal, Pakistan, Thailand, and Vietnam (Chen and Anderberg 2011). It is known for its medicinal properties and is valued in traditional Chinese medicine (Zheng et al. 2015). Notably, it exhibits anticarcinogenic and antibacterial effects, making it valuable in the fight against cancer and bacterial infections. Moreover, the roots of *Duhaldea cappa* are known for their potent anti-inflammatory and immunomodulatory properties (Kalola et al. 2017). However, previous studies on *Duhaldea cappa* mainly focused on examining its chemical constituents and evaluating its pharmacological effects (Zheng et al. 2015). To date, the chloroplast genome of *Duhaldea cappa* has not been reported and analyzed. Therefore, we present the complete chloroplast genome of *Duhaldea cappa*. Analyzing and reporting the chloroplast genome of *Duhaldea cappa* will establish a solid theoretical and data foundation for the efficient development, conservation, and utilization of this species.

## Materials and methods

**Figure 1.**
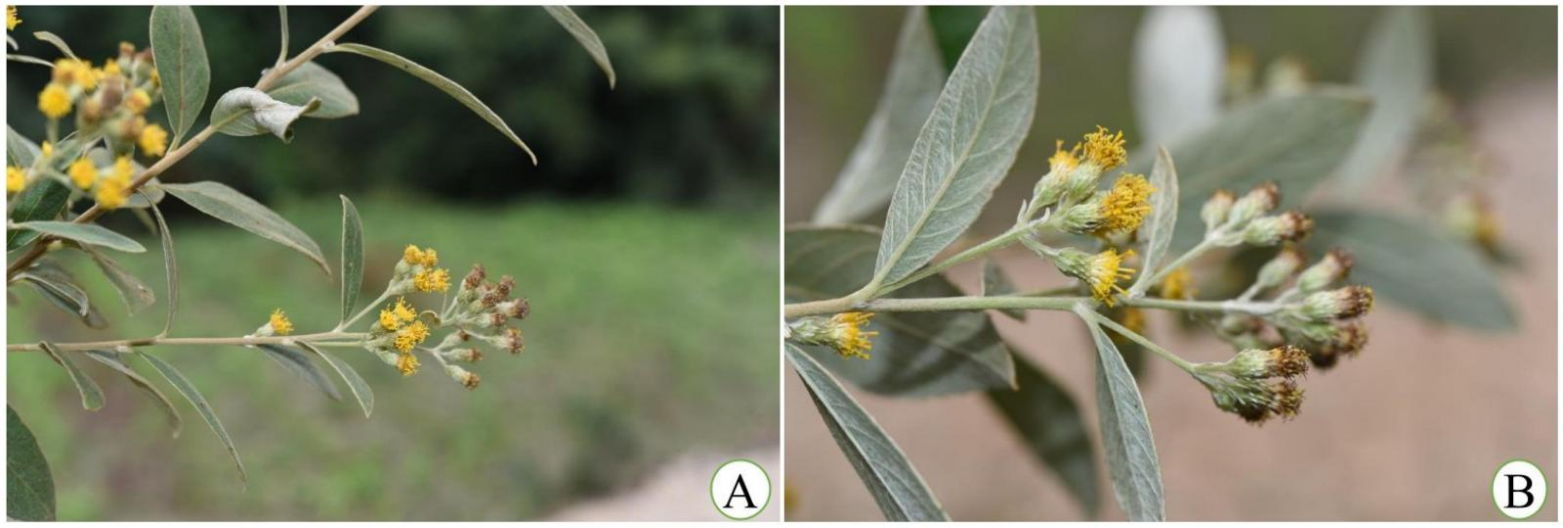
Photographs of (A) flowering branches and (B) capitulum of *Duhaldea cappa. Duhaldea cappa* is a shrub, 70–200 cm tall; stems lanate-tomentose, branched; leaf blade elliptic, lanceolate, or narrowly oblong; capitula radiate or disciform, in dense corymbs. Photos were taken by Dr. Zhixi Fu in Chuxiong city, Yunnan province, China (101°29′41.28″E, 25°1′33.59″N), August 2020 without any copyright issues.

The sample of *Duhaldea cappa* was collected from Shanqian Town, Chuxiong City, Yunnan Province, China (101°29′41.28″E, 25°1′33.59″N) (Figure 1), and the voucher specimen was deposited at the herbarium of the Sichuan Normal University, China (SCNU) (https://bio.sicnu.edu.cn/; contact person: Dr. Zhixi Fu, email: fuzx2017@sicnu.edu.cn) under the voucher number: Junjia Luo 088. Using a modified CTAB method (Allen et al. 2006), we successfully extracted total genomic DNA from the leaves of *Duhaldea cappa*, and the extracted DNA was then sequenced on an Illumina HiSeq XTen platform (San Diego, CA, USA). The raw data was mapped to the reference sequences (MN974527), generating in a BAM format file. From the BAM files, the paired reads were extracted. These paired reads were subsequently assembled using SPAdes (Bankevich et al. 2012), resulting in a FASTG format file. The generated FASTG file was visualized using Bandage (Wick et al. 2015). The sequencing depth was calculated using samtools depth, and the mean values were plotted at intervals of 2000 bp. Subsequently, the results were annotated by PGA (Qu et al. 2019). The annotation results were checked using Geneious R11 (Kearse et al. 2012). The cis- and trans-splicing genes were detected by the program CPGview (Liu et al. 2023). Using the same software, the circular gene map of the *Duhaldea cappa* plastid genome was visualized. We performed nucleobase content and genes analysis on the platform JSHYClound (www.jshycloud.net). The genome sequence of *Duhaldea cappa* has been deposited in GenBank (accession number: NC068630 and OM457000).

In order to understand the phylogenetic relationship of *Duhaldea cappa*, 22 complete chloroplast genomes were downloaded from GenBank to build phylogenetic relationship. The maximum likelihood (ML) phylogenetic tree was constructed based on 23 complete chloroplast genomes, including *Anthriscus cerefolium* (family Apiaceae) and *Kalopanax septemlobus* as the outgroups. Firstly, the concatenated file was aligned by the program MAFFT 7.409 program (Katoh and Standley 2013) using default settings. Then, a molecular phylogenetic tree was generated by the GTR＋GAMMA model in RAxML (Stamatakis et al. 2008) with 1000 bootstrap replicates.

## Results

**Figure 2.**
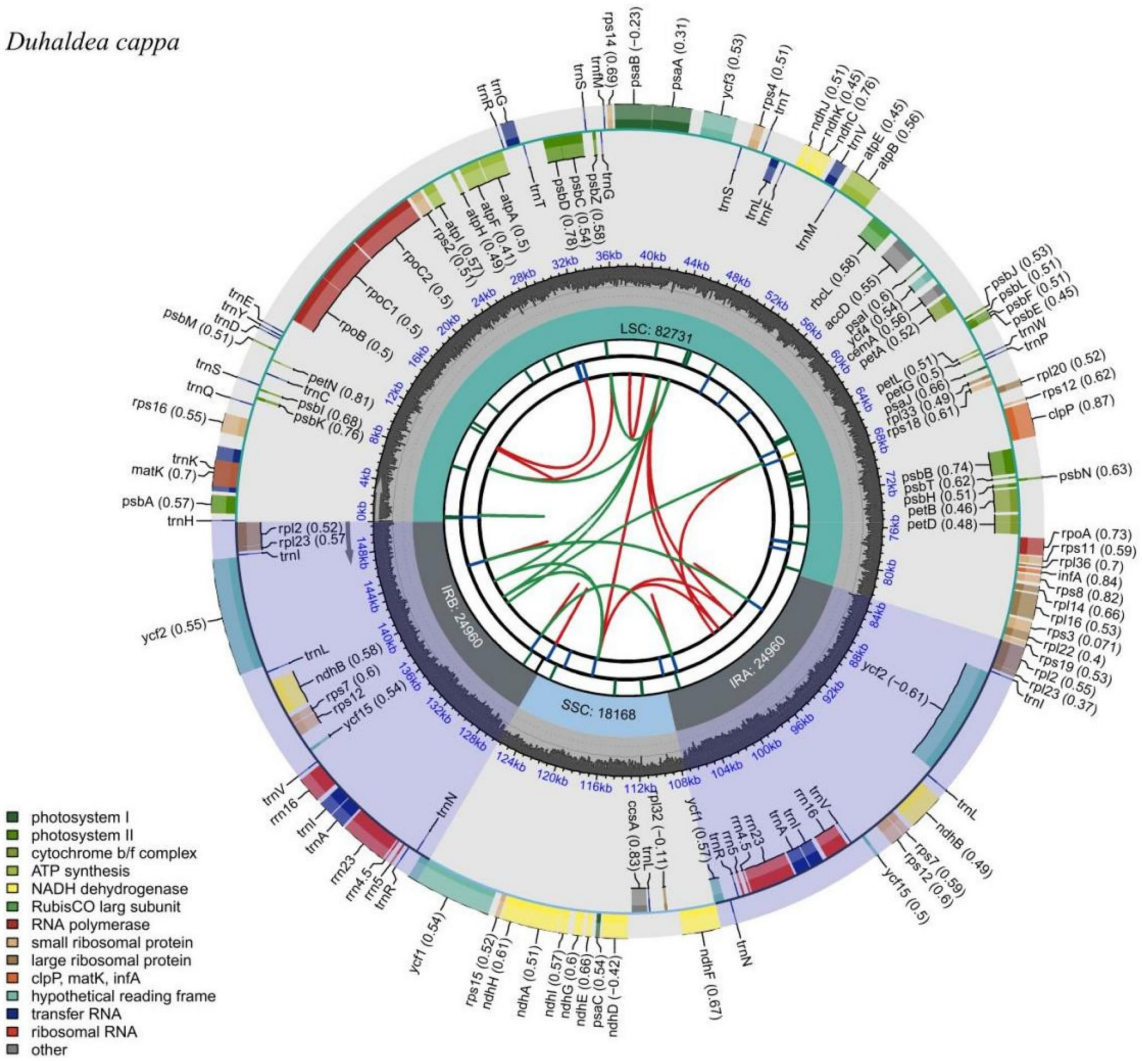
Genomic map of overall features of *Duhaldea cappa* chloroplast genome, generated by CPGview. The species’ name is shown in the top left corner. The map contains six tracks by default. From the center outward, the first track shows the dispersed repeats. The dispersed repeats consist of direct (D) and palindromic (P) repeats, connected with red and green arcs. The second track shows the long tandem repeats as short blue bars. The third track shows the short tandem repeats or microsatellite sequences as short bars with different colors. The colors, the type of repeat they represent, and the description of the repeat types are as follows. The small single-copy (SSC), inverted repeat (IRa and IRb), and large single-copy (LSC) regions are shown on the fourth track. The GC content along the genome is plotted on the fifth track. The genes are shown on the sixth track. The optional codon usage bias is displayed in the parenthesis after the gene name. Genes are color-coded by their functional classification. The transcription directions for the inner and outer genes are clockwise and anticlockwise, respectively. The functional classification of the genes is shown in the bottom left corner.

**Figure 3.**
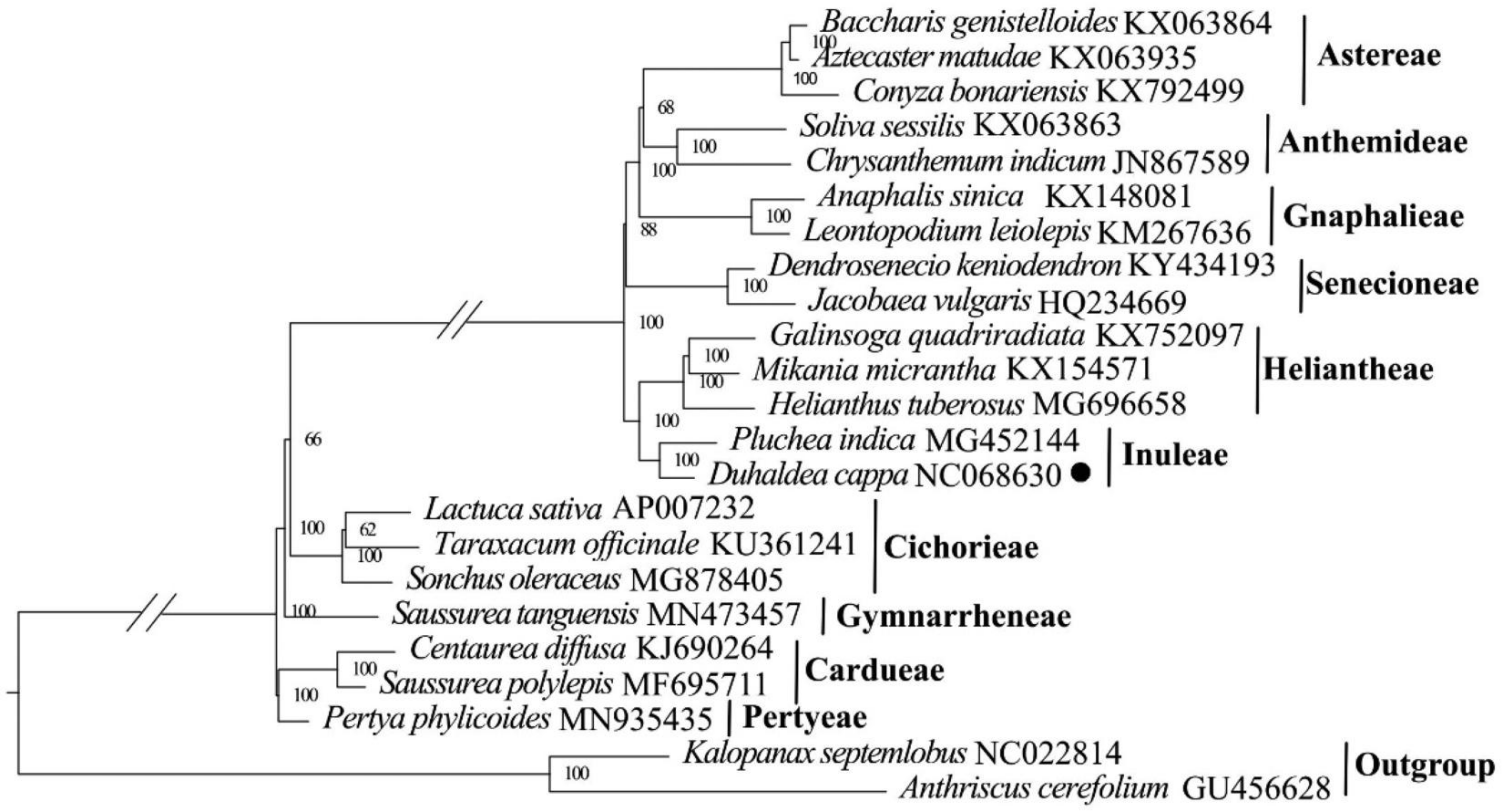
Maximum-likelihood phylogeny of *Duhaldea cappa* and related taxa based on 23 complete chloroplast genomes. The numbers on the branches represent the bootstrap values based on 1000 replicates. The sequences used for tree construction are as follows: *Baccharis genistelloides* (KX063864; Vargas et al. 2017), *Aztecaster matudae* (KX063935; Vargas et al. 2017), *Conyza bonariensis* (KX792499; Wang et al. 2018), *Leontopodium leiolepi*s (KM267636), *Anaphalis sinica* (KX148081), *Chrysanthemum indicum* (JN867589), *Soliva sessilis* (KX063863; Vargas et al. 2017), *Jacobaea vulgaris* (HQ234669; Doorduin et al. 2011), *Dendrosenecio keniodendron* (KY434193), *Galinsoga quadriradiata* (KX752097; Wang et al. 2018), *Mikania micrantha* (KX154571; Huang et al. 2016), *Helianthus tuberosus* (MG696658), *Pluchea indica* (MG452144; Zhang et al. 2017), *Lactuca sativa* (AP007232), *Taraxacum officinale* (KU361241), *Sonchus oleraceus* (MG878405; Hereward et al. 2018), *Saussurea tanguensis* (MN573457), *Saussurea polylepis* (MF695711; Yun et al. 2017), *Centaurea diffusa* (KJ690264), *Pertya phylicoides* (MN935435; Wang et al. 2020), *Anthriscus cerefolium* (GU456628; Downie and Jansen 2015), *Kalopanax septemlobus* (NC022814; Li et al. 2013). The circle represents newly sequenced species (*Duhaldea cappa* genbank No. NC068630).

The total length of the chloroplast genome of *Duhaldea cappa* is 150,819 bp, including one large single copy (LSC) region (82,731 bp), one small single copy (SSC) region (18,168 bp), and two inverted repeat (IR) regions (24,960 bp) (Figure 2). The GC content of the complete chloroplast genome is 37.72%, with 43.05%, 35.91%, and 31.34% in the IR, LSC and SSC regions, respectively. Totally, 133 genes were identified in the genome of *Duhaldea cappa*, including 88 protein-coding genes, 37 tRNA genes, and 8 rRNA genes. Among them, 19 genes were repeated in IR regions, including 8 protein-coding genes (*ndhB*, *rpl2*, *rpl23*, *rps12*, *rps7*, *ycf1*, *ycf15*, *ycf2*), 7 tRNA genes (*trnA*-*UGC*, *trnI*-*CAU*, *trnI*-*GAU*, *trnL*-*CAA*, *trnN*-*GUU*, *trnR*-*ACG*, *trnV*-*GAC*) and 4 rRNA genes (*rrn16*, *rrn23*, *rrn4*.5, *rrn5*). 16 genes (*ndhA*, *ndhB*, *petB*, *petD*, *atpF*, *rpl16*, *rpl2*, *rps12*, *rps16*, *rpoC1*, *trnA*-*UGC*, *trnG*-*UCC*, *trnI*-*GAU*, *trnK*-*UUU*, *trnL*-*UAA*, *trnV*-*UAC*) contained one intron, while *clpP*, *ycf3*, and *rps12* possessed two introns. ML phylogenetic tree consistently showed a close relationship between *Duhaldea cappa* and *Pluchea indica* (MG452144) with a high bootstrap value of 100 (Figure 3). The chloroplast genome of *Duhaldea cappa* was correctly assembled according to the coverage depth (Supplementary Figure 1). The maps of the annotated chloroplast genome, cis-splicing genes, and trans-splicing genes of *Duhaldea cappa* are displayed in Supplementary Figure 2.

## Discussion and conclusion

In this study, we report the complete chloroplast genome and reconstruct the phylogenetic relationship of *Duhaldea cappa*. The results showed that the complete chloroplast genomes of *Duhaldea cappa* possessed a typical quadripartite structure. The result is similar to other species of family Asteraceae (Chen et al. 2018, Kim et al. 2020). The phylogenetic study reconstructed from complete plastomes indicated that *Duhaldea cappa* is closely related to *Pluchea indica* within the tribe Inuleae. The study provides baseline genomic information of *Duhaldea cappa*.

## Supplementary Material

Supplemental Material

## Data Availability

After uploading the data, the NCBI database releases two accession numbers of *Duhaldea cappa*. These two accession numbers contain exactly the same sequence information and author. Data are available in the NCBI GenBank at https://www.ncbi.nlm.nih.gov (accession number: NC068630 and OM457000). The associated BioProject, SRA, and Bio-Sample numbers are PRJNA938187, SRR23606895 and SAMN33427715, respectively.
