# Supplementary material for: Characterization and phylogenetic analysis of the chloroplast genome of Duhaldea cappa (Buch.-Ham. ex D.Don) Pruski & Anderb. (Asteraceae)
Source: Mitochondrial DNA B Resour. 2024 Jan 25;9(1):186–90. doi: 10.1080/23802359.2024.2306203 (PMC10812854; doi:10.1080/23802359.2024.2306203)
Supplement: Supplemental Material [file TMDN_A_2306203_SM2950.docx]

**Complete chloroplast genome features and phylogenetic analysis of *Duhaldea cappa* (Asteraceae: Inuleae)**

**Junjia Luo^a,b^, Xiaofeng Liu^b^, Tingyu Li^b^, Hui Chen^b^, Tianmeng Qu^b^, Yueguang Wang^b^, Shuhua Yu^b#^ , Zhixi Fu^a, b, c#^**

a Key Laboratory of Land Resources Evaluation and Monitoring in Southwest (Sichuan Normal University), Ministry of Education, Chengdu, China;

b College of Life Sciences, Sichuan Normal University, Chengdu, China

c Sustainable Development Research Center of Resources and Environment of Western Sichuan, Sichuan Normal University, Chengdu, China.

# Correspondence:

Shuhua Yu

2822142890@qq.com

Zhixi Fu

fuzx2017@sicnu.edu.cn

**
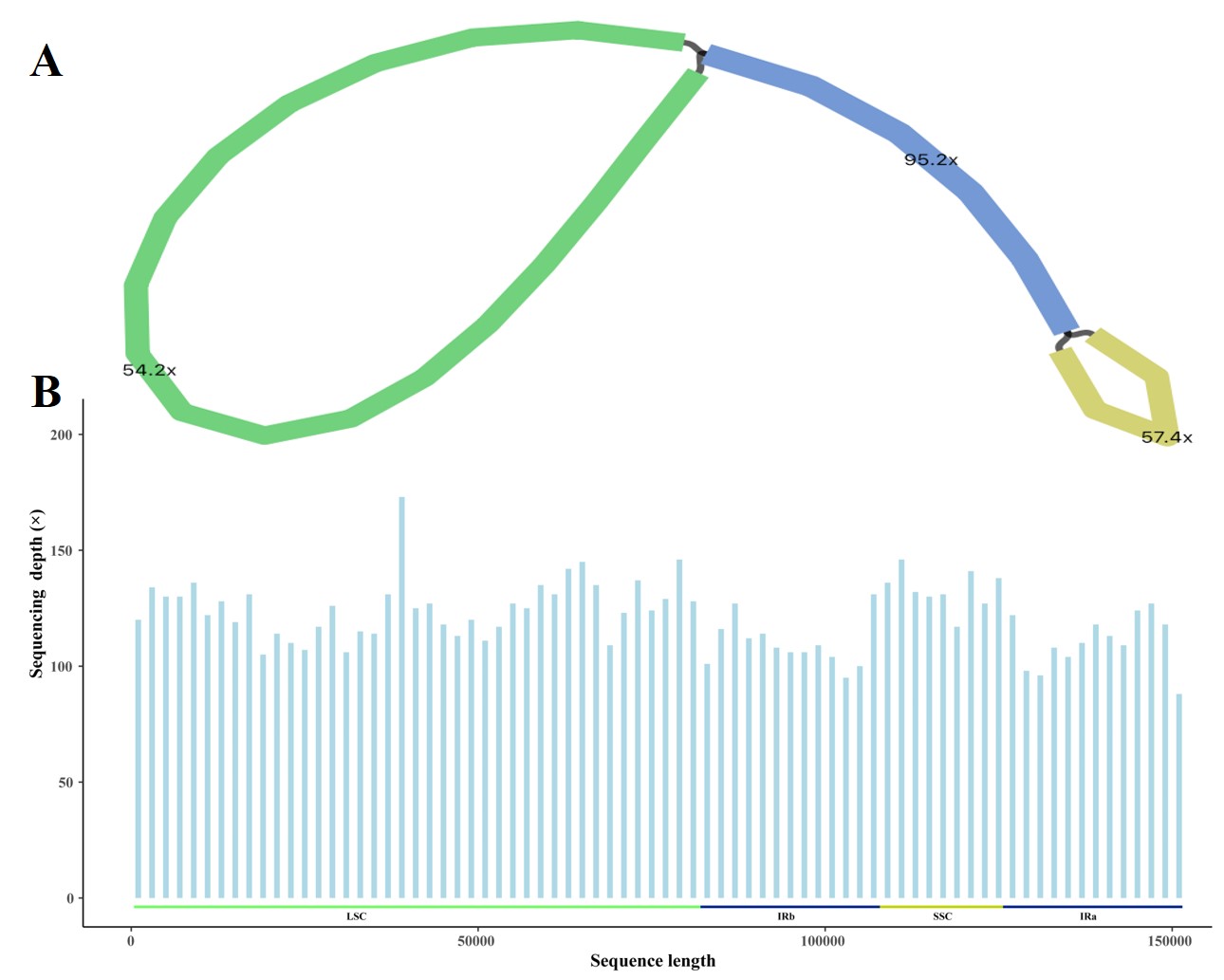
**

**Supplementary Figure 1.** A. The Bandage software to visualise the assembly results of *Duhaldea cappa*. B. The read coverage depth map of *Duhaldea cappa*. The sequencing depth was determined using samtools depth, and the mean values were plotted at intervals of 2000 bp.


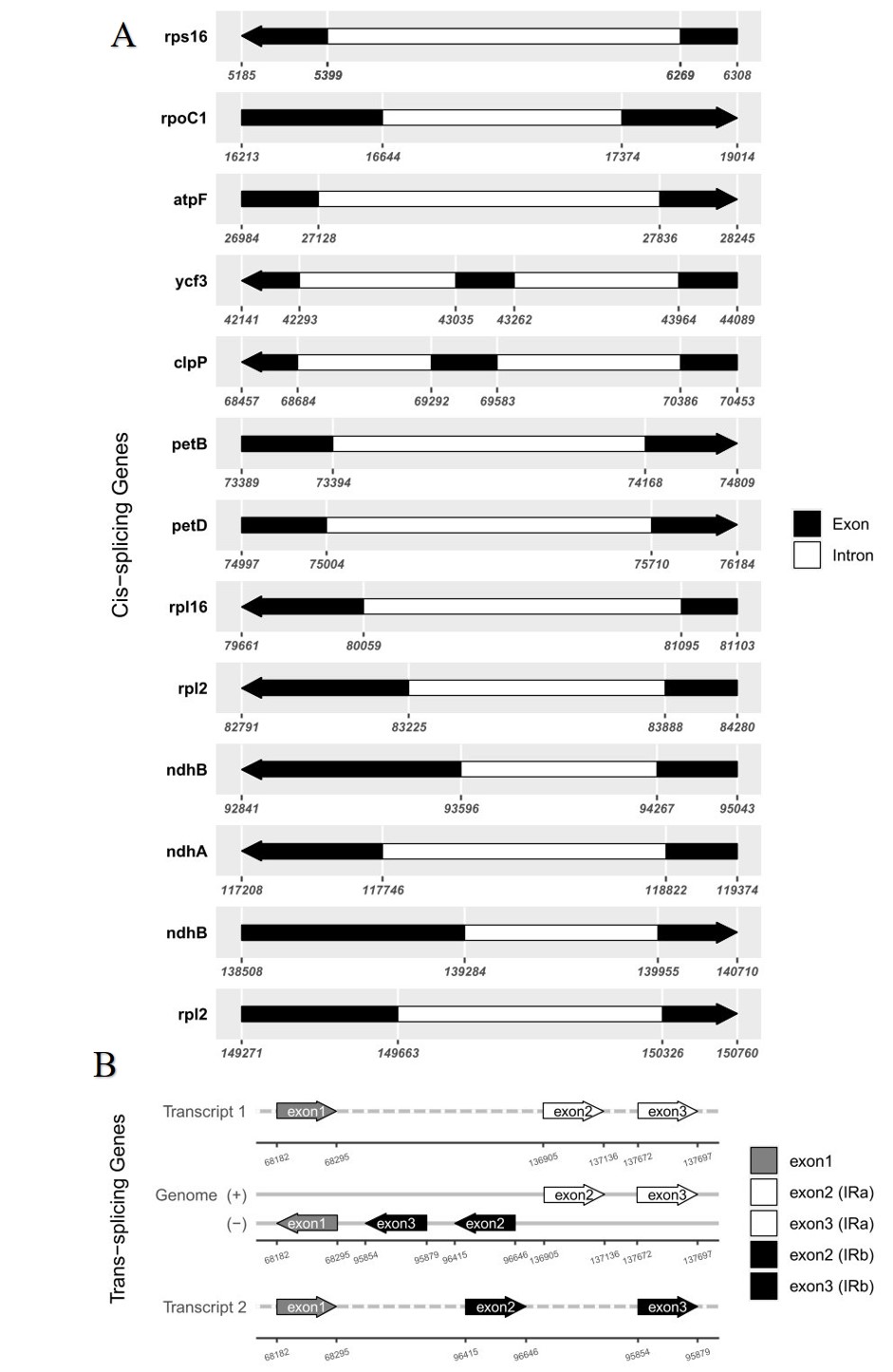


**Supplementary Figure 2.** A. Schematic map of the cis-splicing genes in the *Duhaldea cappa* chloroplast genome. B. Schematic map of the trans-splicing gene *rps12* in the chloroplast genome.
